# 2,2′-[(1*E*)-3-Phenyl­prop-2-ene-1,1-di­yl]bis­(3-hy­droxy-5,5-dimethyl­cyclo­hex-2-en-1-one)

**DOI:** 10.1107/S1600536811033745

**Published:** 2011-08-27

**Authors:** Yu-Lin Zhu, Guo-Lan Xiao, Yan-Fen Chen, Rui-Ting Chen, Ying Zhou

**Affiliations:** a School of Chemistry and Environment, South China Normal University, Guangzhou 510006, People’s Republic of China

## Abstract

In the title mol­ecule, C_25_H_30_O_4_, the two cyclo­hexene rings adopt envelope conformations. The two hy­droxy groups are involved in the formation of intra­molecular O—H⋯O hydrogen bonds. In the crystal structure, weak inter­molecular C—H⋯O hydrogen bonds link mol­ecules related by translation along the axis *a* into chains.

## Related literature

For related structures, see: Bolte *et al.* (2001 ▶); Palakshi Reddy *et al.* (2010 ▶); Shi *et al.* (1998 ▶). For applications of related compounds, see: Ali *et al.* (2011 ▶); Wang *et al.* (2006 ▶). For the synthesis of related compounds, see: Ramachary & Mamillapalli (2007 ▶); Rohr & Mahrwald (2009 ▶).
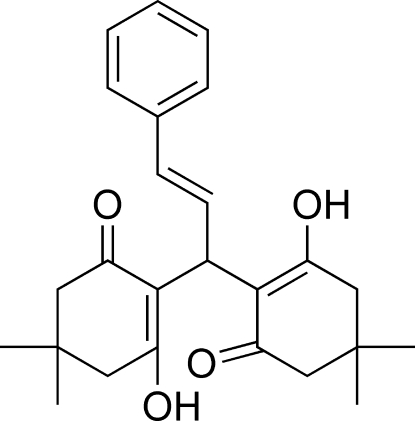


## Experimental

### 

#### Crystal data

C_25_H_30_O_4_
*M* *_r_* = 394.49
Triclinic, 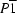

*a* = 5.9465 (15) Å
*b* = 11.214 (3) Å
*c* = 17.170 (4) Å
α = 82.804 (3)°
β = 81.062 (3)°
γ = 76.927 (3)°
*V* = 1096.9 (5) Å^3^
*Z* = 2
Mo *K*α radiation
μ = 0.08 mm^−1^
*T* = 293 K
0.30 × 0.28 × 0.20 mm

#### Data collection

Bruker APEXII area-detector diffractometer
Absorption correction: multi-scan (*SADABS*; Bruker, 2004 ▶) *T*_min_ = 0.977, *T*_max_ = 0.984
11804 measured reflections
4149 independent reflections
3560 reflections with *I* > 2σ(*I*)
*R*_int_ = 0.020

#### Refinement

*R*[*F* ^2^ > 2σ(*F* ^2^)] = 0.071
*wR*(*F* ^2^) = 0.154
*S* = 1.02
4149 reflections
269 parameters
H-atom parameters constrained
Δρ_max_ = 0.29 e Å^−3^
Δρ_min_ = −0.20 e Å^−3^

### 

Data collection: *APEX2* (Bruker, 2004 ▶); cell refinement: *SAINT* (Bruker, 2004 ▶); data reduction: *SAINT*; program(s) used to solve structure: *SHELXS97* (Sheldrick, 2008 ▶); program(s) used to refine structure: *SHELXL97* (Sheldrick, 2008 ▶); molecular graphics: *SHELXTL* (Sheldrick, 2008 ▶); software used to prepare material for publication: *SHELXTL*.

## Supplementary Material

Crystal structure: contains datablock(s) global, I. DOI: 10.1107/S1600536811033745/cv5137sup1.cif

Structure factors: contains datablock(s) I. DOI: 10.1107/S1600536811033745/cv5137Isup2.hkl

Supplementary material file. DOI: 10.1107/S1600536811033745/cv5137Isup3.cml

Additional supplementary materials:  crystallographic information; 3D view; checkCIF report

## Figures and Tables

**Table 1:** Hydrogen-bond geometry (Å, °)

*D*—H⋯*A*	*D*—H	H⋯*A*	*D*⋯*A*	*D*—H⋯*A*
O2—H2⋯O4	0.82	1.82	2.610 (3)	163
O3—H3⋯O1	0.82	1.85	2.640 (3)	160
C19—H19⋯O4^i^	0.93	2.54	3.349 (3)	146
C14—H14*B*⋯O1^ii^	0.97	2.59	3.439 (4)	146

Symmetry codes: (i) 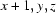; (ii) 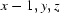.

**Fig. 1.**
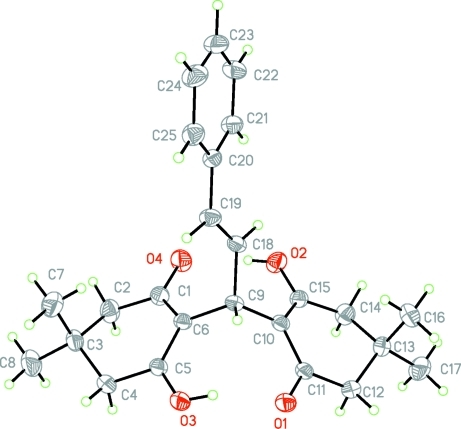
View of (I) showing the atom-labelling scheme. Displacement ellipsoids are drawn at the 50% probability level.

**Fig. 2.**
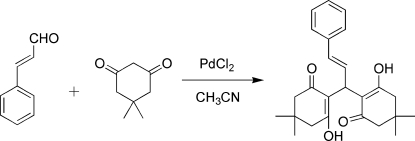
Palladium(II) chloride catalyzed synthesis of the title compound.

## supplementary crystallographic information

### Comment

Tetraketones constitute an important class of organic compounds which can be used as dyes, fluorescent materials for visualization of biomolecules or in laser technologies due to their useful spectroscopic properties (Wang *et al.*, 2006). The title compound is an aldol condensation/Michael addition compound which can be used as an intermediate during the synthesis of oxathene derivatives (Ali *et al.*, 2011). When comes to the unactivated aldehydes such as cinnamaldehyde, the open chain structure can be obtained in a good yield (Ramachary & Mamillapalli, 2007; Rohr & Mahrwald, 2009). The reaction between cinnamaldehyde and 5,5-dimethyl-1,3-cyclohexanedione in the presence palladium(II) chloride proceeded to give the title compound (I) in isolated yield 82%.

In (I) (Fig. 1), the bond lengths and angles are normal and correspond to those observed in related structures (Bolte *et al.*, 2001; Palakshi Reddy *et al.*, 2010; Shi *et al.*, 1998). Two six-membered cyclohexene rings adopt an envelope conformation. The phenyl ring C20–C25 is twisted at 18.1 (1)° from the C18═C19—C20 plane. The hydroxy groups and carbonyl O atoms face each other and form two intramolecular O—H···O hydrogen bonds (Table 1). There are weak intermolecular C19—H19···O4, C14—H14B···O1 and C9—H9···O2 interactions which link molecules into chains.

In the crystal structure, weak intermolecular C—H···O hydrogen bonds (Table 1) link the molecules related by translation along axis *a* into chains.

### Experimental

The title compound has been synthesized following the known procedures (Ramachary & Mamillapalli, 2007; Rohr & Mahrwald, 2009). A mixture of cinnamaldehyde (0.66 g, 5 mmol), 5,5-dimethyl-1,3-cyclohexanedione (1.40 g, 10 mmol), and palladium (II) chloride (0.0010 g) was refluxed in acetonitrile (10 ml) at 353 K for 6 h (Fig. 2). After being cooled to room temperature, the reaction mixture was poured into water. The white precipitate was filtered off with a silica pad, washed twice with water, and the filtrate was then dried under vacuum to yield the product in yield of 82%. Single crystals of the title compound were obtained by slow evaporation from ethanol at room temperature to yield colourless, block-shaped crystals.

### Refinement

The H atoms were positioned geometrically (C—H 0.93–0.98 Å, O—H 0.82 Å) and allowed to ride on their parent atoms, with *U*_iso_ = 1.2 or 1.5U_eq_(parent atom).

### Crystal data

**Table:**

C_25_H_30_O_4_	*Z* = 2
*M**_r_* = 394.49	*F*(000) = 424
Triclinic, *P*1	*D*_x_ = 1.194 Mg m^−^^3^
Hall symbol: -P 1	Mo *K*α radiation, λ = 0.71073 Å
*a* = 5.9465 (15) Å	Cell parameters from 2118 reflections
*b* = 11.214 (3) Å	θ = 2.3–27.2°
*c* = 17.170 (4) Å	µ = 0.08 mm^−^^1^
α = 82.804 (3)°	*T* = 293 K
β = 81.062 (3)°	Block, colourless
γ = 76.927 (3)°	0.30 × 0.28 × 0.20 mm
*V* = 1096.9 (5) Å^3^	

### Data collection

**Table:**

Bruker APEXII area-detector diffractometer	4149 independent reflections
Radiation source: fine-focus sealed tube	3560 reflections with *I* > 2σ(*I*)
graphite	*R*_int_ = 0.020
φ and ω scans	θ_max_ = 26.0°, θ_min_ = 1.9°
Absorption correction: multi-scan (*SADABS*; Bruker, 2004)	*h* = −7→6
*T*_min_ = 0.977, *T*_max_ = 0.984	*k* = −13→13
11804 measured reflections	*l* = −21→9

### Refinement

**Table:**

Refinement on *F*^2^	Secondary atom site location: difference Fourier map
Least-squares matrix: full	Hydrogen site location: inferred from neighbouring sites
*R*[*F*^2^ > 2σ(*F*^2^)] = 0.071	H-atom parameters constrained
*wR*(*F*^2^) = 0.154	*w* = 1/[σ^2^(*F*_o_^2^) + (0.019*P*)^2^ + 1.9414*P*] where *P* = (*F*_o_^2^ + 2*F*_c_^2^)/3
*S* = 1.02	(Δ/σ)_max_ < 0.001
4149 reflections	Δρ_max_ = 0.29 e Å^−^^3^
269 parameters	Δρ_min_ = −0.20 e Å^−^^3^
0 restraints	Extinction correction: *SHELXL97* (Sheldrick, 2008), Fc^*^=kFc[1+0.001xFc^2^λ^3^/sin(2θ)]^-1/4^
Primary atom site location: structure-invariant direct methods	Extinction coefficient: 0.0130 (19)

### Special details

**Table:**

Geometry. All esds (except the esd in the dihedral angle between two l.s. planes) are estimated using the full covariance matrix. The cell esds are taken into account individually in the estimation of esds in distances, angles and torsion angles; correlations between esds in cell parameters are only used when they are defined by crystal symmetry. An approximate (isotropic) treatment of cell esds is used for estimating esds involving l.s. planes.
Refinement. Refinement of F^2^ against ALL reflections. The weighted R-factor wR and goodness of fit S are based on F^2^, conventional R-factors R are based on F, with F set to zero for negative F^2^. The threshold expression of F^2^ > 2sigma(F^2^) is used only for calculating R-factors(gt) etc. and is not relevant to the choice of reflections for refinement. R-factors based on F^2^ are statistically about twice as large as those based on F, and R- factors based on ALL data will be even larger.

### Fractional atomic coordinates and isotropic or equivalent isotropic displacement parameters (Å^2^)

**Table:**

	*x*	*y*	*z*	*U*_iso_*/*U*_eq_	
C6	0.2857 (5)	0.8045 (2)	0.81379 (16)	0.0357 (6)	
C5	0.4316 (5)	0.7081 (2)	0.85206 (16)	0.0374 (6)	
C1	0.0541 (5)	0.8394 (3)	0.85021 (17)	0.0429 (7)	
C4	0.3647 (6)	0.6518 (3)	0.93287 (17)	0.0506 (8)	
H4A	0.5010	0.6299	0.9604	0.061*	
H4B	0.3156	0.5764	0.9283	0.061*	
C2	−0.0248 (6)	0.7860 (3)	0.93157 (19)	0.0576 (9)	
H2A	−0.1034	0.7208	0.9267	0.069*	
H2B	−0.1377	0.8494	0.9586	0.069*	
C3	0.1700 (6)	0.7341 (3)	0.98281 (17)	0.0514 (8)	
C7	0.2611 (8)	0.8383 (4)	1.0080 (2)	0.0740 (11)	
H7A	0.3860	0.8043	1.0389	0.111*	
H7B	0.1375	0.8896	1.0392	0.111*	
H7C	0.3169	0.8863	0.9619	0.111*	
C8	0.0803 (8)	0.6583 (4)	1.0562 (2)	0.0821 (13)	
H8A	0.0167	0.5950	1.0405	0.123*	
H8B	−0.0386	0.7107	1.0883	0.123*	
H8C	0.2064	0.6215	1.0861	0.123*	
C9	0.3718 (4)	0.8650 (2)	0.73423 (15)	0.0342 (6)	
H9	0.5418	0.8459	0.7315	0.041*	
C20	0.3317 (5)	1.2099 (2)	0.75974 (16)	0.0379 (6)	
C18	0.3039 (5)	1.0046 (2)	0.72762 (16)	0.0398 (6)	
H18	0.1955	1.0426	0.6938	0.048*	
C19	0.3858 (5)	1.0746 (2)	0.76568 (18)	0.0427 (7)	
H19	0.4901	1.0350	0.8005	0.051*	
C21	0.1371 (5)	1.2795 (3)	0.72830 (18)	0.0458 (7)	
H21	0.0347	1.2401	0.7110	0.055*	
C25	0.4806 (6)	1.2720 (3)	0.78509 (18)	0.0482 (7)	
H25	0.6132	1.2275	0.8060	0.058*	
C23	0.2417 (7)	1.4659 (3)	0.7482 (2)	0.0605 (9)	
H23	0.2115	1.5514	0.7444	0.073*	
C24	0.4348 (7)	1.3986 (3)	0.7797 (2)	0.0593 (9)	
H24	0.5354	1.4387	0.7975	0.071*	
C22	0.0926 (6)	1.4064 (3)	0.7221 (2)	0.0573 (9)	
H22	−0.0381	1.4516	0.7004	0.069*	
C10	0.3235 (5)	0.8101 (2)	0.66340 (15)	0.0355 (6)	
C11	0.4927 (5)	0.7122 (2)	0.63160 (16)	0.0395 (6)	
C15	0.1190 (5)	0.8469 (3)	0.62977 (16)	0.0404 (6)	
C12	0.4642 (6)	0.6603 (3)	0.55792 (18)	0.0521 (8)	
H12A	0.3949	0.5890	0.5732	0.063*	
H12B	0.6169	0.6329	0.5288	0.063*	
C13	0.3140 (6)	0.7512 (3)	0.50335 (17)	0.0494 (7)	
C14	0.0882 (5)	0.8041 (3)	0.55416 (18)	0.0523 (8)	
H14A	0.0015	0.8728	0.5234	0.063*	
H14B	−0.0049	0.7418	0.5664	0.063*	
C16	0.4406 (7)	0.8516 (3)	0.4663 (2)	0.0679 (10)	
H16A	0.3454	0.9085	0.4320	0.102*	
H16B	0.5847	0.8157	0.4362	0.102*	
H16C	0.4721	0.8941	0.5072	0.102*	
C17	0.2593 (8)	0.6843 (4)	0.4381 (2)	0.0787 (12)	
H17A	0.1771	0.6215	0.4618	0.118*	
H17B	0.4020	0.6474	0.4077	0.118*	
H17C	0.1646	0.7421	0.4040	0.118*	
O3	0.6399 (3)	0.65715 (19)	0.81993 (12)	0.0469 (5)	
H3	0.6525	0.6755	0.7719	0.070*	
O1	0.6739 (4)	0.66073 (19)	0.66454 (12)	0.0501 (5)	
O2	−0.0650 (4)	0.9210 (2)	0.66144 (13)	0.0554 (6)	
H2	−0.0526	0.9260	0.7078	0.083*	
O4	−0.1002 (4)	0.9153 (2)	0.81522 (13)	0.0588 (6)	

### Atomic displacement parameters (Å^2^)

**Table:**

	*U*^11^	*U*^22^	*U*^33^	*U*^12^	*U*^13^	*U*^23^
C6	0.0404 (14)	0.0296 (13)	0.0385 (14)	−0.0068 (11)	−0.0109 (11)	−0.0022 (10)
C5	0.0417 (15)	0.0331 (13)	0.0377 (14)	−0.0070 (11)	−0.0087 (11)	−0.0025 (11)
C1	0.0365 (15)	0.0466 (16)	0.0442 (16)	−0.0036 (12)	−0.0084 (12)	−0.0052 (13)
C4	0.063 (2)	0.0411 (16)	0.0410 (16)	−0.0011 (14)	−0.0069 (14)	0.0042 (13)
C2	0.0456 (18)	0.076 (2)	0.0470 (18)	−0.0086 (16)	−0.0009 (14)	−0.0033 (16)
C3	0.0582 (19)	0.0573 (19)	0.0348 (15)	−0.0079 (15)	−0.0048 (13)	0.0016 (13)
C7	0.090 (3)	0.076 (3)	0.058 (2)	−0.006 (2)	−0.020 (2)	−0.0202 (19)
C8	0.094 (3)	0.092 (3)	0.046 (2)	−0.009 (2)	0.003 (2)	0.008 (2)
C9	0.0320 (13)	0.0282 (12)	0.0403 (14)	−0.0028 (10)	−0.0063 (11)	0.0007 (10)
C20	0.0396 (14)	0.0348 (14)	0.0373 (14)	−0.0053 (11)	−0.0014 (11)	−0.0055 (11)
C18	0.0470 (16)	0.0309 (13)	0.0404 (15)	−0.0041 (12)	−0.0124 (12)	0.0014 (11)
C19	0.0408 (15)	0.0347 (14)	0.0519 (17)	0.0003 (12)	−0.0159 (13)	−0.0048 (12)
C21	0.0474 (17)	0.0377 (15)	0.0522 (17)	−0.0039 (13)	−0.0116 (13)	−0.0069 (13)
C25	0.0503 (17)	0.0484 (17)	0.0492 (17)	−0.0129 (14)	−0.0095 (14)	−0.0087 (14)
C23	0.082 (3)	0.0341 (16)	0.063 (2)	−0.0151 (17)	0.0035 (18)	−0.0068 (15)
C24	0.070 (2)	0.0486 (18)	0.066 (2)	−0.0238 (17)	−0.0063 (18)	−0.0157 (16)
C22	0.064 (2)	0.0383 (16)	0.062 (2)	0.0031 (15)	−0.0098 (17)	0.0021 (14)
C10	0.0381 (14)	0.0300 (13)	0.0369 (14)	−0.0073 (11)	−0.0034 (11)	0.0013 (10)
C11	0.0435 (15)	0.0321 (13)	0.0374 (14)	−0.0052 (12)	0.0009 (12)	0.0043 (11)
C15	0.0406 (15)	0.0373 (14)	0.0420 (15)	−0.0073 (12)	−0.0036 (12)	−0.0031 (12)
C12	0.069 (2)	0.0375 (15)	0.0447 (17)	−0.0006 (14)	−0.0048 (15)	−0.0065 (13)
C13	0.063 (2)	0.0474 (17)	0.0367 (15)	−0.0073 (15)	−0.0066 (14)	−0.0069 (13)
C14	0.0488 (18)	0.061 (2)	0.0493 (18)	−0.0090 (15)	−0.0148 (14)	−0.0093 (15)
C16	0.082 (3)	0.063 (2)	0.049 (2)	−0.0109 (19)	−0.0005 (18)	0.0112 (16)
C17	0.106 (3)	0.076 (3)	0.055 (2)	−0.002 (2)	−0.024 (2)	−0.0255 (19)
O3	0.0456 (12)	0.0445 (11)	0.0429 (11)	0.0047 (9)	−0.0072 (9)	0.0005 (9)
O1	0.0460 (12)	0.0466 (12)	0.0470 (12)	0.0077 (9)	−0.0030 (9)	−0.0001 (9)
O2	0.0422 (12)	0.0646 (14)	0.0536 (13)	0.0089 (10)	−0.0110 (10)	−0.0155 (11)
O4	0.0404 (12)	0.0707 (15)	0.0545 (13)	0.0098 (11)	−0.0093 (10)	−0.0009 (11)

### Geometric parameters (Å, °)

**Table:**

C6—C5	1.388 (4)	C21—H21	0.9300
C6—C1	1.411 (4)	C25—C24	1.378 (4)
C6—C9	1.515 (4)	C25—H25	0.9300
C5—O3	1.308 (3)	C23—C24	1.371 (5)
C5—C4	1.485 (4)	C23—C22	1.377 (5)
C1—O4	1.272 (3)	C23—H23	0.9300
C1—C2	1.498 (4)	C24—H24	0.9300
C4—C3	1.528 (4)	C22—H22	0.9300
C4—H4A	0.9700	C10—C15	1.383 (4)
C4—H4B	0.9700	C10—C11	1.413 (4)
C2—C3	1.529 (4)	C11—O1	1.280 (3)
C2—H2A	0.9700	C11—O1	1.280 (3)
C2—H2B	0.9700	C11—C12	1.504 (4)
C3—C8	1.524 (5)	C15—O2	1.304 (3)
C3—C7	1.527 (5)	C15—C14	1.490 (4)
C7—H7A	0.9600	C12—C13	1.524 (4)
C7—H7B	0.9600	C12—H12A	0.9700
C7—H7C	0.9600	C12—H12B	0.9700
C8—H8A	0.9600	C13—C16	1.514 (5)
C8—H8B	0.9600	C13—C14	1.523 (4)
C8—H8C	0.9600	C13—C17	1.536 (4)
C9—C18	1.520 (3)	C14—H14A	0.9700
C9—C10	1.522 (4)	C14—H14B	0.9700
C9—H9	0.9800	C16—H16A	0.9600
C20—C21	1.385 (4)	C16—H16B	0.9600
C20—C25	1.391 (4)	C16—H16C	0.9600
C20—C19	1.472 (4)	C17—H17A	0.9600
C18—C19	1.297 (4)	C17—H17B	0.9600
C18—H18	0.9300	C17—H17C	0.9600
C19—H19	0.9300	O3—H3	0.8200
C21—C22	1.381 (4)	O2—H2	0.8200
			
C5—C6—C1	117.4 (3)	C20—C21—H21	119.4
C5—C6—C9	120.6 (2)	C24—C25—C20	121.0 (3)
C1—C6—C9	122.0 (2)	C24—C25—H25	119.5
O3—C5—C6	123.2 (2)	C20—C25—H25	119.5
O3—C5—C4	113.6 (2)	C24—C23—C22	119.7 (3)
C6—C5—C4	123.2 (3)	C24—C23—H23	120.2
O4—C1—C6	122.0 (3)	C22—C23—H23	120.2
O4—C1—C2	116.4 (3)	C23—C24—C25	120.3 (3)
C6—C1—C2	121.6 (3)	C23—C24—H24	119.8
C5—C4—C3	114.5 (2)	C25—C24—H24	119.8
C5—C4—H4A	108.6	C23—C22—C21	120.0 (3)
C3—C4—H4A	108.6	C23—C22—H22	120.0
C5—C4—H4B	108.6	C21—C22—H22	120.0
C3—C4—H4B	108.6	C15—C10—C11	117.5 (3)
H4A—C4—H4B	107.6	C15—C10—C9	124.0 (2)
C1—C2—C3	114.7 (3)	C11—C10—C9	118.5 (2)
C1—C2—H2A	108.6	O1—C11—C10	122.4 (3)
C3—C2—H2A	108.6	O1—C11—C10	122.4 (3)
C1—C2—H2B	108.6	O1—C11—C12	116.5 (2)
C3—C2—H2B	108.6	O1—C11—C12	116.5 (2)
H2A—C2—H2B	107.6	C10—C11—C12	121.1 (3)
C8—C3—C7	109.4 (3)	O2—C15—C10	123.4 (3)
C8—C3—C4	109.6 (3)	O2—C15—C14	113.8 (3)
C7—C3—C4	110.1 (3)	C10—C15—C14	122.9 (3)
C8—C3—C2	110.1 (3)	C11—C12—C13	113.9 (2)
C7—C3—C2	110.4 (3)	C11—C12—H12A	108.8
C4—C3—C2	107.3 (3)	C13—C12—H12A	108.8
C3—C7—H7A	109.5	C11—C12—H12B	108.8
C3—C7—H7B	109.5	C13—C12—H12B	108.8
H7A—C7—H7B	109.5	H12A—C12—H12B	107.7
C3—C7—H7C	109.5	C16—C13—C14	111.0 (3)
H7A—C7—H7C	109.5	C16—C13—C12	109.8 (3)
H7B—C7—H7C	109.5	C14—C13—C12	106.9 (3)
C3—C8—H8A	109.5	C16—C13—C17	109.7 (3)
C3—C8—H8B	109.5	C14—C13—C17	109.4 (3)
H8A—C8—H8B	109.5	C12—C13—C17	110.0 (3)
C3—C8—H8C	109.5	C15—C14—C13	114.8 (3)
H8A—C8—H8C	109.5	C15—C14—H14A	108.6
H8B—C8—H8C	109.5	C13—C14—H14A	108.6
C6—C9—C18	113.8 (2)	C15—C14—H14B	108.6
C6—C9—C10	114.3 (2)	C13—C14—H14B	108.6
C18—C9—C10	113.0 (2)	H14A—C14—H14B	107.5
C6—C9—H9	104.8	C13—C16—H16A	109.5
C18—C9—H9	104.8	C13—C16—H16B	109.5
C10—C9—H9	104.8	H16A—C16—H16B	109.5
C21—C20—C25	117.8 (3)	C13—C16—H16C	109.5
C21—C20—C19	122.3 (3)	H16A—C16—H16C	109.5
C25—C20—C19	119.9 (3)	H16B—C16—H16C	109.5
C19—C18—C9	124.9 (3)	C13—C17—H17A	109.5
C19—C18—H18	117.6	C13—C17—H17B	109.5
C9—C18—H18	117.6	H17A—C17—H17B	109.5
C18—C19—C20	127.1 (3)	C13—C17—H17C	109.5
C18—C19—H19	116.4	H17A—C17—H17C	109.5
C20—C19—H19	116.4	H17B—C17—H17C	109.5
C22—C21—C20	121.1 (3)	C5—O3—H3	109.5
C22—C21—H21	119.4	C15—O2—H2	109.5
			
C1—C6—C5—O3	171.1 (3)	C19—C20—C25—C24	179.5 (3)
C9—C6—C5—O3	−6.2 (4)	C22—C23—C24—C25	0.3 (5)
C1—C6—C5—C4	−7.9 (4)	C20—C25—C24—C23	−0.7 (5)
C9—C6—C5—C4	174.8 (3)	C24—C23—C22—C21	0.4 (5)
C5—C6—C1—O4	−170.8 (3)	C20—C21—C22—C23	−0.6 (5)
C9—C6—C1—O4	6.5 (4)	C6—C9—C10—C15	87.1 (3)
C5—C6—C1—C2	7.5 (4)	C18—C9—C10—C15	−45.1 (4)
C9—C6—C1—C2	−175.2 (3)	C6—C9—C10—C11	−90.3 (3)
O3—C5—C4—C3	159.0 (3)	C18—C9—C10—C11	137.5 (2)
C6—C5—C4—C3	−21.9 (4)	C15—C10—C11—O1	−170.8 (3)
O4—C1—C2—C3	−159.0 (3)	C9—C10—C11—O1	6.7 (4)
C6—C1—C2—C3	22.6 (4)	C15—C10—C11—O1	−170.8 (3)
C5—C4—C3—C8	167.7 (3)	C9—C10—C11—O1	6.7 (4)
C5—C4—C3—C7	−72.0 (4)	C15—C10—C11—C12	7.2 (4)
C5—C4—C3—C2	48.1 (4)	C9—C10—C11—C12	−175.2 (2)
C1—C2—C3—C8	−167.7 (3)	C11—C10—C15—O2	168.1 (3)
C1—C2—C3—C7	71.4 (4)	C9—C10—C15—O2	−9.3 (4)
C1—C2—C3—C4	−48.5 (4)	C11—C10—C15—C14	−11.2 (4)
C5—C6—C9—C18	−135.7 (3)	C9—C10—C15—C14	171.5 (3)
C1—C6—C9—C18	47.1 (3)	O1—C11—C12—C13	−155.9 (3)
C5—C6—C9—C10	92.5 (3)	O1—C11—C12—C13	−155.9 (3)
C1—C6—C9—C10	−84.7 (3)	C10—C11—C12—C13	25.9 (4)
C6—C9—C18—C19	69.0 (4)	C11—C12—C13—C16	69.2 (4)
C10—C9—C18—C19	−158.5 (3)	C11—C12—C13—C14	−51.3 (4)
C9—C18—C19—C20	178.0 (3)	C11—C12—C13—C17	−170.0 (3)
C21—C20—C19—C18	18.1 (5)	O2—C15—C14—C13	162.1 (3)
C25—C20—C19—C18	−160.8 (3)	C10—C15—C14—C13	−18.5 (4)
C25—C20—C21—C22	0.1 (4)	C16—C13—C14—C15	−71.7 (4)
C19—C20—C21—C22	−178.9 (3)	C12—C13—C14—C15	48.0 (4)
C21—C20—C25—C24	0.5 (4)	C17—C13—C14—C15	167.0 (3)

### Hydrogen-bond geometry (Å, °)

**Table:**

*D*—H···*A*	*D*—H	H···*A*	*D*···*A*	*D*—H···*A*
O2—H2···O4	0.82	1.82	2.610 (3)	163.
O3—H3···O1	0.82	1.85	2.640 (3)	160.
C19—H19···O4^i^	0.93	2.54	3.349 (3)	146.
C14—H14B···O1^ii^	0.97	2.59	3.439 (4)	146.

Symmetry codes: (i) *x*+1, *y*, *z*; (ii) *x*−1, *y*, *z*.
